# A New Thiopeptide Antibiotic, Micrococcin P3, from a Marine-Derived Strain of the Bacterium *Bacillus stratosphericus*

**DOI:** 10.3390/molecules25194383

**Published:** 2020-09-24

**Authors:** Weihong Wang, Kyu-Hyung Park, Jusung Lee, Eunseok Oh, Chanyoon Park, Eunmo Kang, Juni Lee, Heonjoong Kang

**Affiliations:** 1 Laboratory of Marine Drugs, School of Earth and Environmental Sciences, Seoul National University, NS-80, Seoul 08826, Korea; pharmacy2007@naver.com (W.W.); grekhp@snu.ac.kr (K.-H.P.); leejusung@snu.ac.kr (J.L.); dmstjr0130@snu.ac.kr (E.O.); owun3@snu.ac.kr (E.K.); lgl5278@snu.ac.kr (J.L.); 2 Research Institute of Oceanography, Seoul National University, NS-80, Seoul 08826, Korea; 3 Interdisciplinary Graduate Program in Genetic Engineering, Seoul National University, NS-80, Seoul 08826, Korea; chanyoon0126@snu.ac.kr

**Keywords:** *Bacillus stratosphericus*, thiopeptide, antibiotic, antibacterial activity

## Abstract

A new thiopeptide (micrococcin P3, **1**) and a known one (micrococcin P1, **2**) were isolated from the culture broth of a marine-derived strain of *Bacillus stratosphericus*. The structures of both compounds were elucidated using spectroscopic methods, including extensive 1D and 2D NMR analysis, high resolution mass spectrometry (HRMS), and tandem mass spectrometry. Both compounds exhibited potent antibacterial activities against Gram-positive strains with minimum inhibitory concentration (MIC) values of 0.05−0.8 μg/mL and did not show cytotoxicity in the MTT assay up to a concentration of 10 μM. This study adds a new promising member, micrococcin P3, to the family of thiopeptide antibiotics, which shows potential for the development of new antibiotics targeting Gram-positive bacteria.

## 1. Introduction

Natural product-derived antibiotics have provided humankind with an effective weapon to fend off bacterial infections and diseases since their discovery in 1929. However, the popularity of antibiotics, especially broad-spectrum antibiotics, has quickly led to their overuse and misuse in people and animals, which has accelerated the emergence and development of antibiotic resistance in pathogens. Increasing resistance to antibiotics reduces the treatment options available for patients and imposes significant economic burdens on patients due to prolonged stays in hospitals. Moreover, medical procedures, such as major surgery and cancer chemotherapy, would become very risky without effective antibiotics to prevent and treat infections. According to the World Health Organization [1], antibiotic resistance is starting to complicate the combat against HIV and malaria as well. Thus, antibiotic resistance is a major global healthcare concern. In this context, it is more critical now than ever to discover new and effective antibiotics to overcome this worldwide public health crisis.

To discover new antibiotics, natural product researchers have dedicated much time and effort to explore novel bio-resources in various environments, including seas. The seas are highly complicated ecosystems with surprising microbial abundances of 10^6^ per mL seawater and up to 10^9^ per mL ocean-bottom sediments. As early as the 1950s, marine bacteria were found to produce antimicrobial compounds. In the 1970s, the first report on the antibiotic properties of marine bacterial metabolites was published [2]. Thereafter, the rate of discovery of new antibiotics from marine microbes has increased significantly with the improvement of structure characterization and sampling technologies. Today, with the application of genetic information-based exploration of natural chemodiversity, marine microbes can still be regarded as excellent sources of new antibiotics. 

During our ongoing program toward the discovery of new antibiotics, 32 marine-derived bacterial strains were screened by MIC (minimum inhibitory concentration) bioassays and surveyed by LC-MS (liquid chromatography-mass spectrometry) screening. Three extracts of bacterial strains displayed antibacterial activities in our MIC bioassays. In one of these antibacterially active extracts (from *Bacillus stratosphericus* 16L088-2), a number of new thiopeptides, albeit in very low amounts, were detected. The solvent partition of the extract followed by repeated chromatography revealed a new thiopeptide, micrococcin P3 (**1**), along with a known one (micrococcin P1, **2**). Thiopeptides are a family of naturally occurring peptide antibiotics with a complex molecular architecture [3]. Distinctive features of the thiopeptide structure include multiple thiazole rings and a highly substituted pyridine centerpiece, or a reduced form thereof, at the junction of the characteristic macrocyclic nucleus [4]. To date, as many as over 100 members of the thiopeptide family have been obtained, but none of them have reached the clinic for humans due to their low aqueous solubility and unfavorable pharmacokinetic properties. However, the profound chemical diversity and potent activities still render thiopeptides attractive in both the chemical and biological arenas. Herein, the isolation, structure elucidation, and antibacterial activity of micrococcins P1 and P3 are described.

## 2. Results and Discussion

### 2.1. Structural Elucidation of the Compounds

Micrococcin P3 (**1**), a white amorphous powder, was determined to have a molecular formula of C_48_H_49_N_13_O_9_S_6_ by high resolution electrospray ionization mass spectrometry (ESIMS) data (*m/z* 1144.2185, calc. for [M + H]^+^ 1144.2179). The UV spectrum showed thiocillin-like absorptions [5] at 220, 289, and 350 nm. The UV spectral data, together with the presence of six sulfur atoms in the highly unsaturated molecule, suggested that the compound is a member of the class of thiopeptides. The ^1^H NMR spectrum (Table 1) revealed deshielded singlets assignable to six thiazoles (Thia, *δ* 8.13, 8.29, 8.32, 8.43, 8.46, and 8.59) and two doublets typical of a 2,3,6-trisubstituted pyridine ring (*δ* 8.32 and 8.45), indicating that compound **1** is a member of series *d* thiopeptides [3]. Other deshielded proton signals were assigned to the six N-bonded protons (*δ* 7.94, 7.98, 8.21, 8.69, 9.54 and 9.85). The remaining signals displayed in the ^1^H NMR spectrum are attributed to seven methyls, one shielded methylene, six methines attached to heteroatoms (O or N), three O-bonded protons, and two olefinics. The ^13^C and edited HSQC (heteronuclear single-quantum correlation spectroscopy) spectra showed the presence of six carbonyl carbons and 15 non-protonated aromatic carbons, 12 of which were derived from disubstituted thiazoles and the other three were assigned to 2,3,6-trisubstituted pyridine. Analysis of COSY (correlation spectroscopy) data established the spin systems of one valine (V), one 2-hydroxypropylamide (Hpa), two threonine (T), and two didehydrobutyrine (Dhb) residues. The sequence of amino acids was determined by interpreting the key HMBC (heteronuclear multiple-bond correlation spectroscopy) correlations, especially those from the nonexchangeable amide protons to their surrounding carbonyl carbons. Additional support for the sequence came from the fragmentation patterns observed in the tandem mass spectra. The sequence Thia^1^-Dhb^1^-Hpa for the C-terminal amino acid side chain was traced from the fragments at *m*/*z* 986 and 1069 (Figure S19).

A literature survey on series *d* thiopeptides revealed a close similarity of compound **1** with micrococcin P1 (**2**), a representative member of the class of thiopeptides. The general features of the ^1^H NMR spectrum of compound **1** are analogous to those of **2** [6], except for those assigned to the didehydrobutyrine residue (Dhb^2^) adjacent to a thiazole (Thia^5^). Beyond our initial exception, the atomic connections of the Dhb^2^-Thia^5^ moiety are exactly the same as those corroborated by the COSY, HSQC and HMBC data. The structure was finally identified by analysis of the ROESY data of compounds **1** and **2**. The NOE correlation between H-8 and H-9 showed the same intensity for both compounds, while the NOE correlation between H-44 and H-45 was not observed at all for compound **1**, but was clearly observed for compound **2** (Figure S2). These observations indicated a 42*E* configuration for the Dhb-Thia^5^ unit of compound **1**, which is different from the 42*Z* configuration of **2** [7].

**Figure 1:**
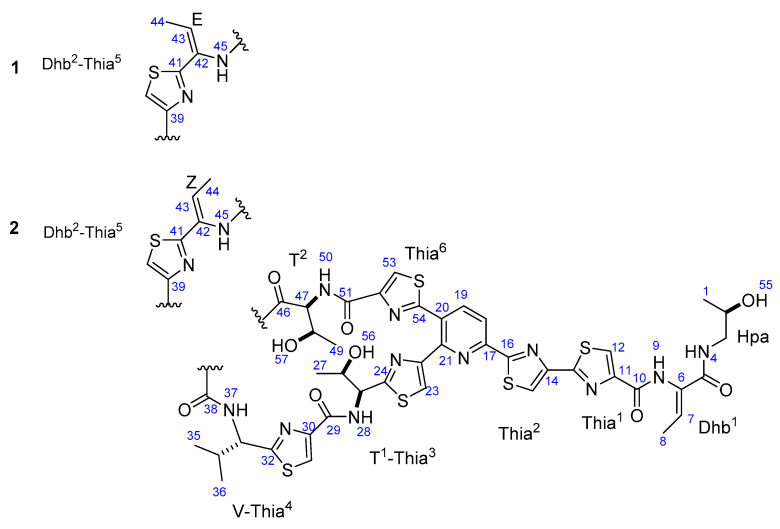
Structures of compounds **1** and **2**.

Although the structures harbored several typical characteristics of a nonribosomal peptide, thiopeptide antibiotics have been reported to be ribosomally synthesized peptides [5,8,9]. The complex molecular architectures of the thiopeptides were concluded to arise from posttranslational modifications of a linear precursor peptide, the composition of which is confined to the 20 proteinogenic amino acids. Thus, **1** was presumed to occur in its natural configuration as shown in Figure 1. This assumption was supported by comparison of the optical rotation and NMR data of **1** with those of **2**, the configuration of which was confirmed by total synthesis [6,10].

### 2.2. Antibacterial Activity

The biological activity of the compounds was evaluated against a panel of terrestrial and marine-derived bacteria using the two fold microtiter broth dilution method [11]. The terrestrial test bacteria, routinely employed in our MIC assays, included three Gram-positive strains (*Staphylococcus aureus* KCTC 1927, *Kocuria rhizophila* KCTC 1915, and *Bacillus subtilis* KCTC 1021) and three Gram-negative strains (*Escherichia coli* KCTC 2441, *Klebsiella pneumoniae* KCTC 2690, and *Salmonella typhimurium* KCTC 2515). Both **1** and **2** strongly inhibited the growth of all three Gram-positive strains with MIC values of 0.05–0.8 μg/mL (Table 2), whereas no antibacterial activity was observed against Gram-negative strains up to a concentration of 26 μg/mL. The different susceptibility of Gram-positive and Gram-negative strains to antibiotics may stem from the structure differences of their cell envelop. Gram-positive bacteria lack an outer membrane but are surrounded by layer of peptidoglycan much thicker than that of Gram-negative strains. The thick peptidoglycan layer may absorb antibiotics more easily than thin one. On the contrary, the outer membrane of Gram-negative strains may function as an evolving barrier against the penetration of antibiotics. The compounds showed no cytotoxicity in the MTT (3-(4,5-dimethylthiazolyl-2)-2,5-diphenyltetrazolium bromide) assay at a concentration of 10 µM. Thus, the new bioactive compound **1** was added to the family of thiopeptide antibiotics and showed potential for the development of new antibiotics targeting Gram-positive bacteria.

This is the first report on the characterization and bioactivities of secondary metabolites from the bacterium *B. stratosphericus*. However, *B. stratosphericus* is a versatile and capable bacterium. A recent example is the hydrocarbonoclastic strain FLU5 of *B. stratosphericus*, which produces an efficient surface-active agent, BS-FLU5, in the presence of various substrates, including residual frying oil [12]. In a previous study, strain FLU5 was also shown to degrade fluoranthene [13], a member of the U.S. Environmental Protection Agency’s 16 priority pollutant polycyclic aromatic hydrocarbon (PAHs). *B. stratosphericus* RNCM B-11677 and B-11678 were found to produce ethanol from lignocellulosic biomass [14,15]. *B. stratosphericus* LT745897 showed potential for biopesticide development due to its antagonistic activity against bacterial phytopathogens [16]. Our study suggests that *B. stratosphericus* represents an alternative source for the discovery of new antibiotics.

Furthermore, the effects of **1** and **2** on the growth of marine-derived bacteria isolated from the same area as *B. stratosphericus* were investigated. Six representative marine-derived bacteria, including *Vibrio parahaemolyticus*, *Photobacterium damselae*, *Shewanella algae*, *Bacillus amyloliquefaciens ssp*-*plantarum*, and *Pseudomonas stutzeri*, were employed in the MIC bioassay. Both compounds displayed strong inhibitory activities against *Enterococcus faecalis*, exhibiting MIC values (0.06−0.8 μg/mL) superior to those of the positive controls (vancomycin and linezolid). In contrast, the antibacterial activities of **1** and **2** against the remaining five marine-derived bacteria (0.5−8 μg/mL) were lower than those of the positive controls (Table S1).

*B. stratosphericus* is commonly found in high concentrations in the stratosphere, hence the name *stratosphericus*. However, it can also be found in various other environments, such as deep seas, estuaries, and desert soils, due to atmospheric cycling processes. *B. stratosphericus* has developed admirable persistence, such as halo tolerance, to adapt to adverse circumstances. In our salt tolerance study, the strain could grow well on plates and in broth with a sea salt concentration up to 15%. Compounds **1** and **2** exhibited weak inhibitory activities against marine-derived bacteria, indicating that *B. stratosphericus* has evolved smart chemical strategies for interspecific competition to acquire bioecological equivalence in the marine community.

### 2.3. Solubility Issues with Thiopeptides

Like most of the other peptides, the main issue of thiopeptides is their poor aqueous solubility, which largely hinders their application in medicine and antimicrobial treatment. This is exemplified by thiostrepton, one of the most extensively studied thiopeptide antibiotics. Although thiostrepton exerts impressive antibacterial activity via a distinct mode of action from those of current chemotherapeutics, it was approved by the US Food and Drug Administration (FDA) only as an external drug for veterinary use [17]. The lack of formulations of thiostrepton proper for human use originates from its poor water solubility and low bioavailability, which also restricts its application in animals. To overcome the obstacles faced in the applications of thiopeptides, various approaches have been tried by many research groups. For example, modification of nocathiacin I through efficient hydrolysis of the side chain generated a series of water-soluble analogues [18]. Structure-based design targeting the bacterial ribosome applied to thiostrepton led to several simpler derivatives [19]. Among the efforts on obtaining analogs with enhanced aqueous solubility, LFF571 is a noteworthy example [20]. LFF571 was developed by Novartis as a result of medicinal chemistry work on improving solubility and efficacy profile of GE2270A. For long time, LFF571 had been considered as a promising novel antibiotic for the treatment of *Clostridium difficile* infection (CDI). It reaches high colonic concentrations after oral administration and was proved safe and well tolerated in healthy volunteers in phase I trial. A phase II trial, started in 2015, showed higher clinical response rates than vancomycin. However, higher recurrence rates than vancomycin were also reported [21]. Thus, LFF571 was unfortunately discontinued from clinical development for CDI in 2019. Nevertheless, when considering the great endeavor carried out by researchers and the potent activities of thiopeptides, it can be believed that solubility issues with thiopeptides will be finally solved and analogs suitable for the treatment of infections will be discovered.

## 3. Materials and Methods

### 3.1. General Experimental Procedures

The optical rotations were measured in MeOH using a Rudolph Research Autopol III (Hackettstown, NJ, USA). UV spectra were recorded on a Hitachi JP/U-3010 UV spectrophotometer (Tokyo, Japan). All NMR spectra were recorded on a Bruker Ascend 700 spectrometer (Billerica Middlesex County, MA, USA) using DMSO-*d*_6_ as solvent. Chemical shifts were reported with reference to the respective solvent peaks (*δ*_H_ 2.50 and *δ*_C_ 39.52 for DMSO-*d*_6_ methanol-*d*_4_). Electrospray ionization (ESI) source low-resolution mass data were obtained on an Agilent Technologies 6120 quadrupole mass spectrometer (Santa Clara, CA, USA) coupled with an Agilent Technologies 1260 series HPLC. High-resolution mass spectrometric data were collected on a JEOL JMS-700 double-focusing (B/E configuration) instrument (Tokyo, Japan). High-performance liquid chromatography (HPLC) was performed using a Waters HPLC (Milford Worcester County, MA, USA) equipped with a Waters 2998 photodiode array detector and Waters 1525 binary pump. HPLC-grade solvents (Daejeon, Korea) were used for HPLC. NMR solvents were purchased from Cambridge Isotope Laboratories Inc. (Andover, MA, USA).

### 3.2. Strain and Cultivation

The bacterial strain 16L088-2 was identified as a new member of the species *B. stratosphericus* on the basis of a partial 16S rRNA gene sequence using the universal primers 27f and 1492r (99.86% similarity to the sequence of *B. stratosphericus* strain 41KF2a; GenBank Accession No. NR042336). The bacterium was grown on SYP agar (10 g of soluble starch, 4 g of yeast extract, 2 g of peptone, and 15 g of agar dissolved in artificial seawater (sea salt 35 g/L distilled H_2_O)). The seed culture was prepared by inoculating the spores of the strain 16L088-2 into 10 mL of SYP liquid medium in a 50-mL conical tube and maintained in a shaking incubator (220 rpm) at 27 °C for three days. The seed culture (10 mL) was then inoculated into 1 L of SYP liquid medium in a 2.5-L Ultra Yield flask. Production fermentation was carried out under the same conditions as seed cultivation.

### 3.3. Extraction and Isolation of Compounds

In total, 40 L of SYP culture was fermented and 4.2 g of crude extract was harvested by EtOAc extraction. The EtOAc extract was then evaporated in vacuo and subjected to silica MPLC, eluting with a step gradient solvent system of 0 to 50% of MeOH-CH_2_Cl_2_, to obtain 8 fractions (1−8). Fraction 4, eluting with 5% MeOH-CH_2_Cl_2_, contained thiopeptides as detected by LC-MS. Fraction 4 was then was subjected to a semi-preparative RP-HPLC system equipped with a Phenomenex Luna C18 column (5 μm, 100 Å, 250 × 100 mm, UV = 230 nm, 2.0 mL/min) and eluted with 40% CH_3_CN in H_2_O (containing 0.05% TFA). Pure compound **1** (1.1 mg) was obtained as a minor peak following the major peak of compound **2** in the chromatogram (Figure S1). Both compounds **1** and **2** were purified again by HPLC in the same condition and the purity (99%) was checked by evaporative light scattering detector.

Micrococcin P3 (**1**): white, amorphous powder; [α]^20^_D_ −65.4 (*c* 0.24, MeOH:acetone = 2:1); UV (MeOH) *λ*_max_ (log *ε*) 220 (5.02), 349 (4.30); ^1^H NMR and ^13^C NMR data, Table 1; ESIMS *m/z* 1144 [M + H]^+^, 1166 [M + Na]^+^; HRESIMS *m/z* 1144.2185 [M + H]^+^ (calcd. for C_48_H_4__9_N_13_O_9_S_6_, 1144.2179).

Micrococcin P1 (**2**): white, amorphous powder; [α]^20^_D_ −65.9 (*c* 0.83, MeOH:acetone = 2:1); UV (MeOH) *λ*_max_ (log *ε*) 220 (5.05), 349 (4.29); ^1^H NMR, Table S2; ^13^C NMR data, Table S3; ESIMS *m/z* 1144 [M + H]^+^, 1166 [M + Na]^+^.

### 3.4. Antibacterial Activity Assay

The following six microorganisms were obtained from the stock culture collection at the Korean Collection for Type Cultures: *Staphylococcus aureus* KCTC 1927, *Kocuria rhizophila* KCTC 1915, *Bacillus subtilis* KCTC 1021, *Escherichia coli* KCTC 2441, *Klebsiella pneumoniae* KCTC 2690, and *Salmonella typhimurium* KCTC 2515. The antibacterial activity was measured by the 2-fold microtiter broth dilution method. Blank sample (100 μL DMSO only) and 100-μL aliquots of the compounds and positive controls (vancomycin and ampicillin) at a concentration of 410 μg/mL in DMSO were added to different wells of column 1 of a 96-well microtiter plate. The wells of other columns were added with 50 μL of Mueller Hinton broth. Blank sample, the compounds, and positive controls were serially diluted by pipetting 50 μL of the sample in the wells of column 1 into the next wells of column 2, and the wells of column 2 into the next wells of column 3, and so on. Then, each well was inoculated with 50 μL of the test bacteria (5 × 10^5^ cfu/mL). Thus, final concentrations of 205, 103, 51, 26, 13, 6.4, 3.2, 1.6, 0.8, 0.4, 0.2, 0.1, 0.05, 0.025 μg/mL of samples were made containing 50%, 25%, 13%, 6.3%, 3.1%, 1.6%, 0.8% 0.4%, 0.2%, 0.1%, 0.05%, 0.02%, 0.01%, 0.005% DMSO (*v*/*v*), respectively. The plates were incubated under static conditions at 37 °C for 24 h. MIC was measured as the concentration at which no growth was observed.

### 3.5. Cell Viability Assay

Cell viability was measured at 48 h post exposure to DMSO or indicated compounds by assessing the conversion of yellow MTT to purple formazan. In short, the CV-1 cells (1 × 10^4^ cells/well) were cultured in 96-well plates at 37 °C for 24 h, and then each well was treated with DMSO or the compounds at the indicated concentration. After 2 days, 10 μL of MTT solution (5 mg/mL) was added to each well, and the plates were incubated at 37 °C for 3 h. The optical density values were determined at 560 nm by a standard microplate reader (Thermo Varioskan Flash, Waltham, MA, USA).

## 4. Conclusions

A new thiopeptide (**1**) and a known one (**2**) were obtained from a marine-derived strain of *Bacillus stratosphericus*. These compounds strongly inhibited the growth of all three tested Gram-positive strains without significant cytotoxicity up to a concentration of 10 µM, as evidenced by the MTT assay. This study adds a new promising member to the family of thiopeptide antibiotics, showing potential for the development of new antibiotics targeting Gram-positive bacteria.

## Figures and Tables

**Table 1:** ^1^H (700 MHz) and ^13^C (175 MHz) NMR data of compound **1** in DMSO-*d*_6_.

Unit ^a^	No.	*δ*_H_^b^	*δ*_C_^c^	Unit ^a^	No.	*δ*_H_^b^	*δ*_C_^c^
Hpa	1	1.01, d (6.2)	21.2	V-Thia^4^	29		160.5
	2	3.70, sext (6.2)	65.2		30		149.2
	3	3.07, m	46.9		31	8.29, s	124.8
	4	7.94, t (5.7)			32		169.9
Dhb^1^	5		164.3		33	5.12, t (9.3)	55.6
	6		130.6		34	2.57, m	32.0
	7	6.50, q (7.0)	128.0		35	0.98, d (6.3)	18.6
	8	1.69, d (7.0)	13.6		36	0.87, d (6.3)	19.8
	9	9.54, s			37	8.69, d (9.3)	
Thia^1^	10		159.1	Dhb^2^-Thia^5^	38		160.0
	11		150.2		39		147.5
	12	8.46, s			40	8.43, s	125.5
	13		161.4		41		162.1
Thia^2^	14		149.4		42		128.8
	15	8.59, s	121.6		43	6.30, q (7.6)	126.9
	16		168.5		44	2.04, d (7.6)	14.1
Pry	17		149.7		45	9.85, s	
	18	8.32, d (8.1)	118.5	T^2^	46		169.1
	19	8.45, d (8.1)	140.8		47	4.56, dd (8.2, 2.9)	58.1
	20		128.6		48	4.36, br s	67.4
	21		151.0		49	1.28, d (6.0)	20.1
T^1^-Thia^3^	22		152.2		50	7.98, d (8.2)	
	23	8.13, s	121.5	Thia^6^	51		160.0
	24		169.6		52		149.7
	25	5.08, dd (8.3, 6.2)	55.9		53	8.32, s	126.0
	26	4.01, m	67.0		54		164.2
	27	1.02, d (6.2)	20.5	OH	55	4.67, d (4.5)	
	28	8.21, d (8.3)			56	4.80, d (4.6)	
					57	5.40, d (6.7)	

**^a^** Hpa, Dhb, Thia, Pry, T, V, and OH are abbreviations for 2-hydroxypropylamide, didehydrobutyrine, thiazole, pyridine, threonine, valine, and hydroxyl group, respectively. **^b^** Proton multiplicity and coupling constants are in parenthesis. **^c^** The ^13^C NMR shifts were assigned on the basis of analysis of heteronuclear single-quantum correlation spectroscopy (HSQC) and heteronuclear multiple-bond correlation spectroscopy (HMBC) data.

**Table 2:** Antibacterial activities (minimum inhibitory concentration (MIC)) of compounds **1** and **2**.

Bacterium	1 (μg/mL)	2 (μg/mL)	Vancomycin (μg/mL)	Linezolid (μg/mL)	DMSO (*v*/*v*)
*Staphylococcus aureus* KCTC 1927	0.8	0.1	0.2	0.8	6.3%
*Kocuria rhizophila* KCTC 1915	0.2	0.05	0.8	0.4	6.3%
*Bacillus subtilis* KCTC 1021	0.8	0.5	0.05	0.4	6.3%
*Escherichia coli* KCTC 2441	26	26	3.2	3.2	6.3%
*Klebsiella pneumoniae* KCTC 2690	26	26	6.4	3.2	6.3%
*Salmonella typhimurium* KCTC 2515	26	26	3.2	3.2	6.3%

## Supplementary Materials

The following are available online, Figure S1: HPLC chromatogram of micrococcins P1 (**2**) and P3 (**1**). Figure S2: High resolution ESIMS spectra of micrococcin P3 (**1**). Figure S3: The bacterium 16L088-2 cultured on SYP agar. Figure S4: ^1^H NMR spectrum of micrococcin P3 (**1**). Figure S5: ^13^C NMR spectrum of micrococcin P3 (**1**). Figure S6: COSY spectrum of micrococcin P3 (**1**). Figure S7: HSQC spectrum of micrococcin P3 (**1**). Figure S8: HMBC spectrum of micrococcin P3 (**1**). Figure S9: ROESY spectrum of micrococcin P3 (**1**). Figure S10: Expended ROESY spectrum of micrococcin P3 (**1**). Figure S11: ^1^H NMR spectrum of micrococcin P1 (**2**). Figure S12: ^13^C NMR spectrum of micrococcin P1 (**2**). Figure S13: COSY spectrum of micrococcin P1 (**2**). Figure S14: HSQC spectrum of micrococcin P1 (**2**). Figure S15: HMBC spectrum of micrococcin P1 (**2**). Figure S16: HMBC spectrum of micrococcin P1 (**2**). Figure S17: Expended ROESY spectrum of micrococcin P1 (**2**). Figure S18: The effects of micrococcins P3 (**1**) and P1 (**2**) on the viability of CV-1 cells. Figure S19: Key MS/MS fragments observed in the spectra of micrococcins P3 (**1**). Table S1: Antibacterial activities of micrococcins P3 (**1**) and P1 (**2**) against marine-derived bacterial strains. Table S2: ^1^H NMR (700 MHz) data of micrococcin P1 (**2**) in DMSO-*d*_6_. Table S3: ^13^C NMR (175 MHz) data of micrococcin P1 (**2**) in DMSO-*d*_6_.
