# Supplementary material for: A New Thiopeptide Antibiotic, Micrococcin P3, from a Marine-Derived Strain of the Bacterium Bacillus stratosphericus
Source: Molecules. 2020 Sep 24;25(19):4383. doi: 10.3390/molecules25194383 (PMC7582574; doi:10.3390/molecules25194383)
Supplement: Supplementary file 1 [file molecules-25-04383-s001.pdf]

A new thiopeptide antibiotic, micrococcin P3, from a marine-derived strain of the bacterium *Bacillus stratosphericus*

Weihong Wang, Kyu-Hyung Park, Jusung Lee, Eunseok Oh, Chanyoon Park, EunMo Kang, Jun Lee and Heonjoong Kang

Table of Contents

Figure S1. HPLC chromatogram of micrococcins P3 (**1**) and P1 (**2**)…………..…………………………..…..……...…...**S3**
Figure S2. High resolution ESIMS spectra of micrococcin P3 (**1**)……………………………………………...……**S4**
Figure S3. The bacterium 16L088-2 cultured on SYP agar..…………………………………………….……….…..**S5**
Figure S4. ^{1}H NMR spectrum of micrococcin P3 (**1**) …………………………………………………….…….....**S6**
Figure S5. ^{13}C NMR spectrum of micrococcin P3 (**1**) ………………………………………………...……..…..**S7**
Figure S6. COSY spectrum of micrococcin P3 (**1**) …………………………………………………………...…**S8**
Figure S7. HSQC spectrum of micrococcin P3 (**1**) ……………………………………………………….…….…**S9**
Figure S8. HMBC spectrum of micrococcin P3 (**1**) ………...………………………………………….………....**S10**
Figure S9. ROESY spectrum of micrococcin P3 (**1**) ……………………………………………………….…..**S11**
Figure S10. Expended ROESY spectrum of micrococcin P3 (**1**) ……………………………….………...…**S12**
Figure S11. ^{1}H NMR spectrum of micrococcin P1 (**2**)…..…… ………………………………………………….…..**S13**
Figure S12. ^{13}C NMR spectrum of micrococcin P1 (**2**)…… …………………………………………………..**S14**
Figure S13. COSY spectrum of micrococcin P1 (**2**)……………………………………………………….….…**S15**
Figure S14. HSQC spectrum of micrococcin P1 (**2**) ………………………...……………………………….**S16**
Figure S15. HMBC spectrum of micrococcin P1 (**2**)………………………………………………………...….**S17**
Figure S16. ROESY spectrum of micrococcin P1 (**2**)…………………………………………………………...…**S18**
Figure S17. Expended ROESYspectrum of micrococcin P1 (**2**)……………………………….……………**S19**
Figure S18. The effects of micrococcins P3 (**1**) and P1 (**2**) on the viability of CV-1 cells…………………….…………**S20**
Figure S19. Key MS/MS fragments observed in the spectra of micrococcins P3 (**1**)……………………………………**S21**
Table S1. Antibacterial activities of micrococcins P3 (**1**) and P1 (**2**) against marine-derived bacterial strains ……..……**S22**
Table S2. ^{1}H NMR (700 MHz) data of micrococcin P1 (**2**) in DMSO-d_6 (J in Hz) …………………...……….………….**S23**
Table S3. ^{13}C NMR (175 MHz) data of micrococcin P1 (**2**) in DMSO-d_6 (J in Hz) ………………...……….………….**S24**

Figure S1. HPLC chromatogram of micrococcins P1 (**2**) and P3 (**1**)

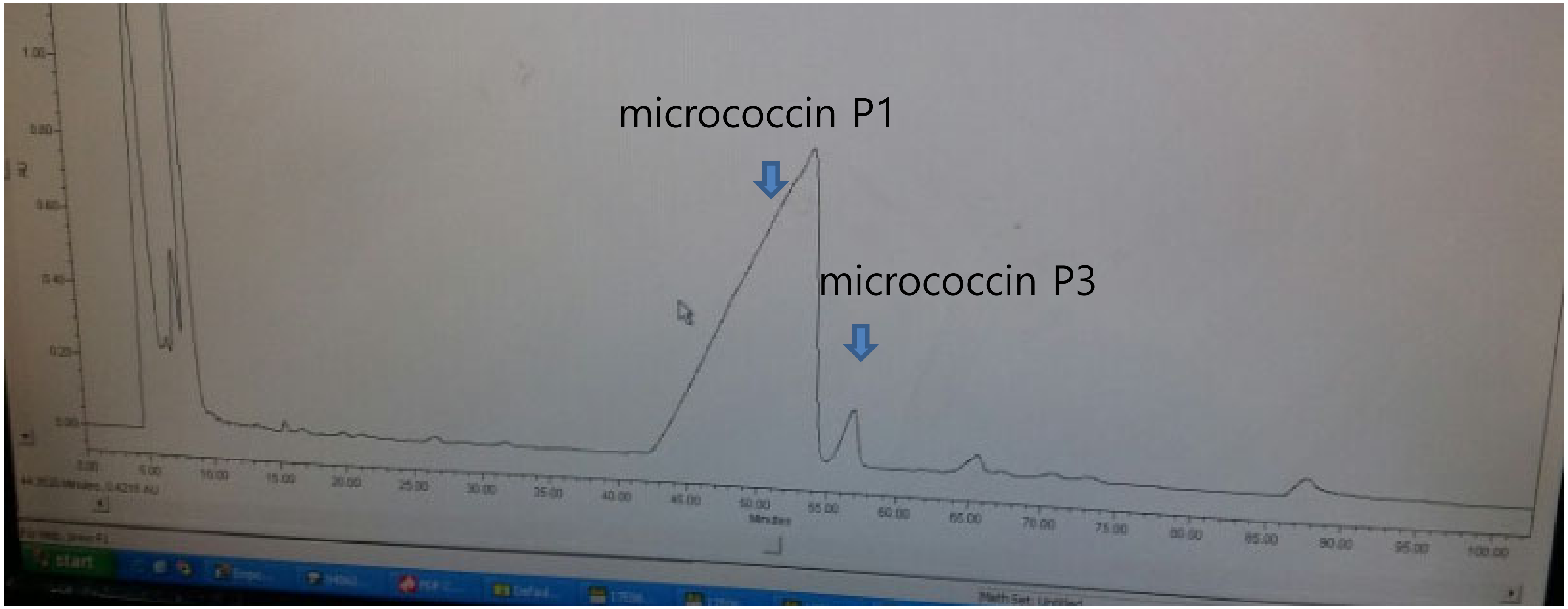

Figure S2. High resolution ESIMS spectra of micrococcin P3 (**1**)

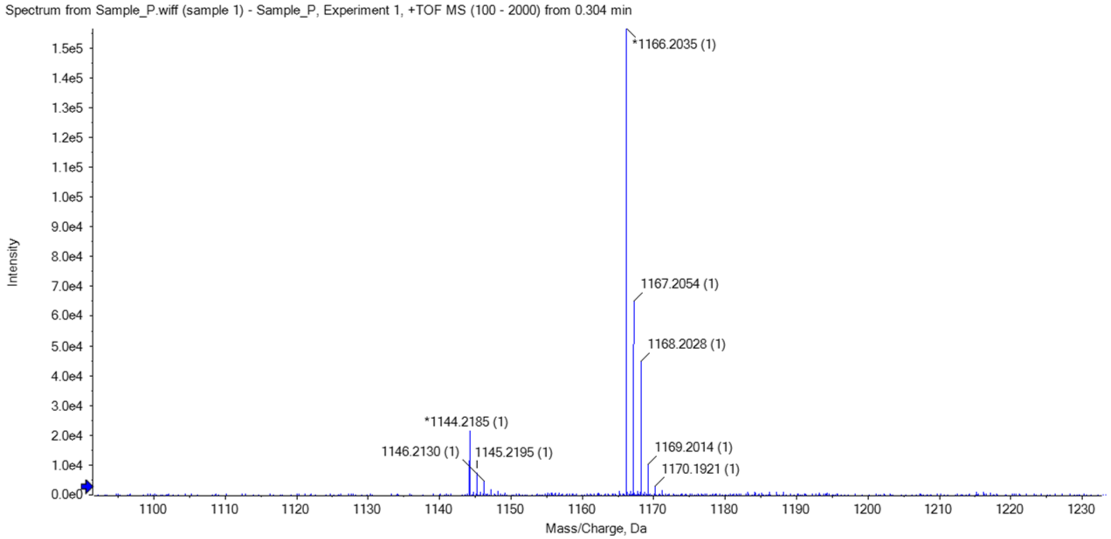

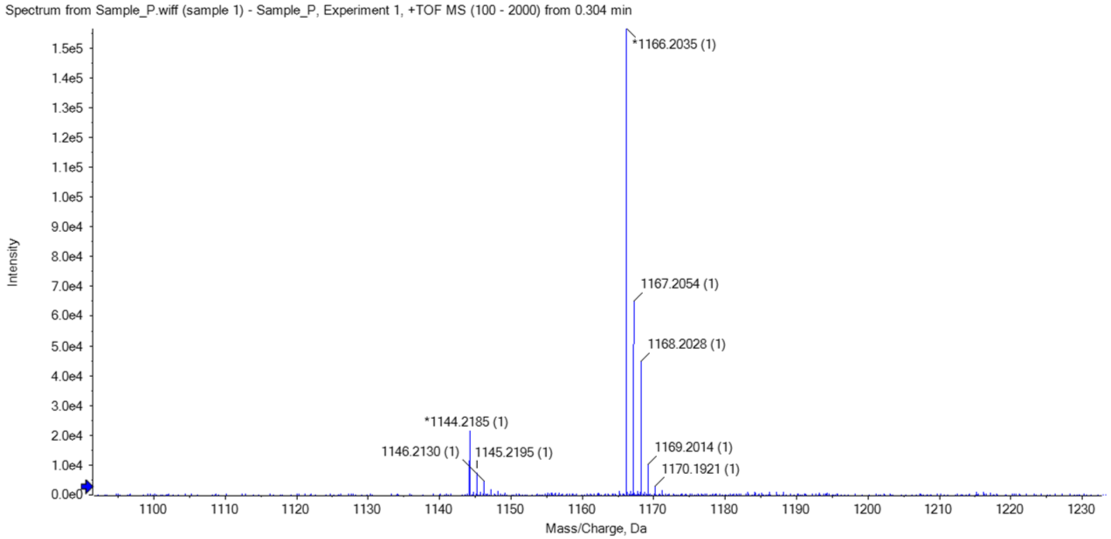

Figure S3. The bacterium 16L088-2 cultured on SYP agar

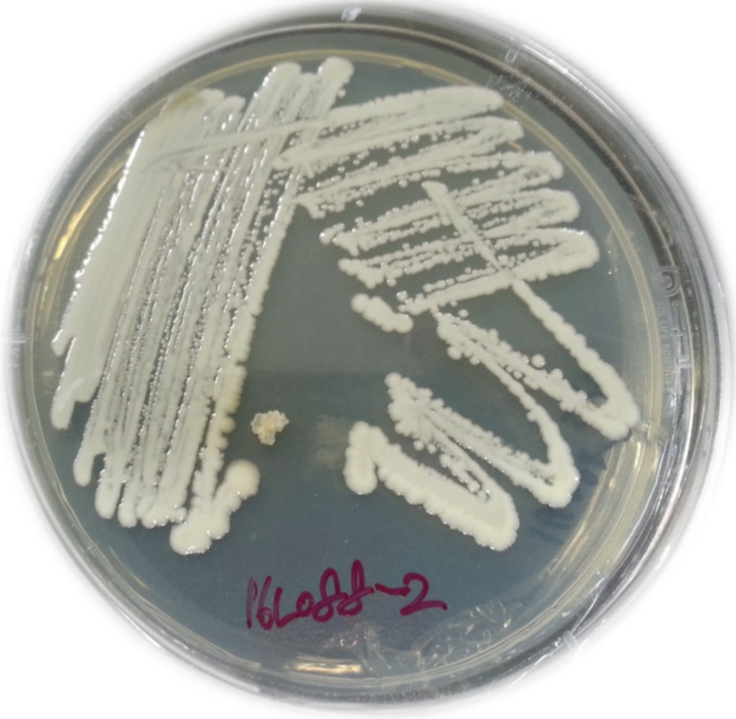

Figure S4. ^{1}H NMR spectrum of micrococcin P3 (**1**)

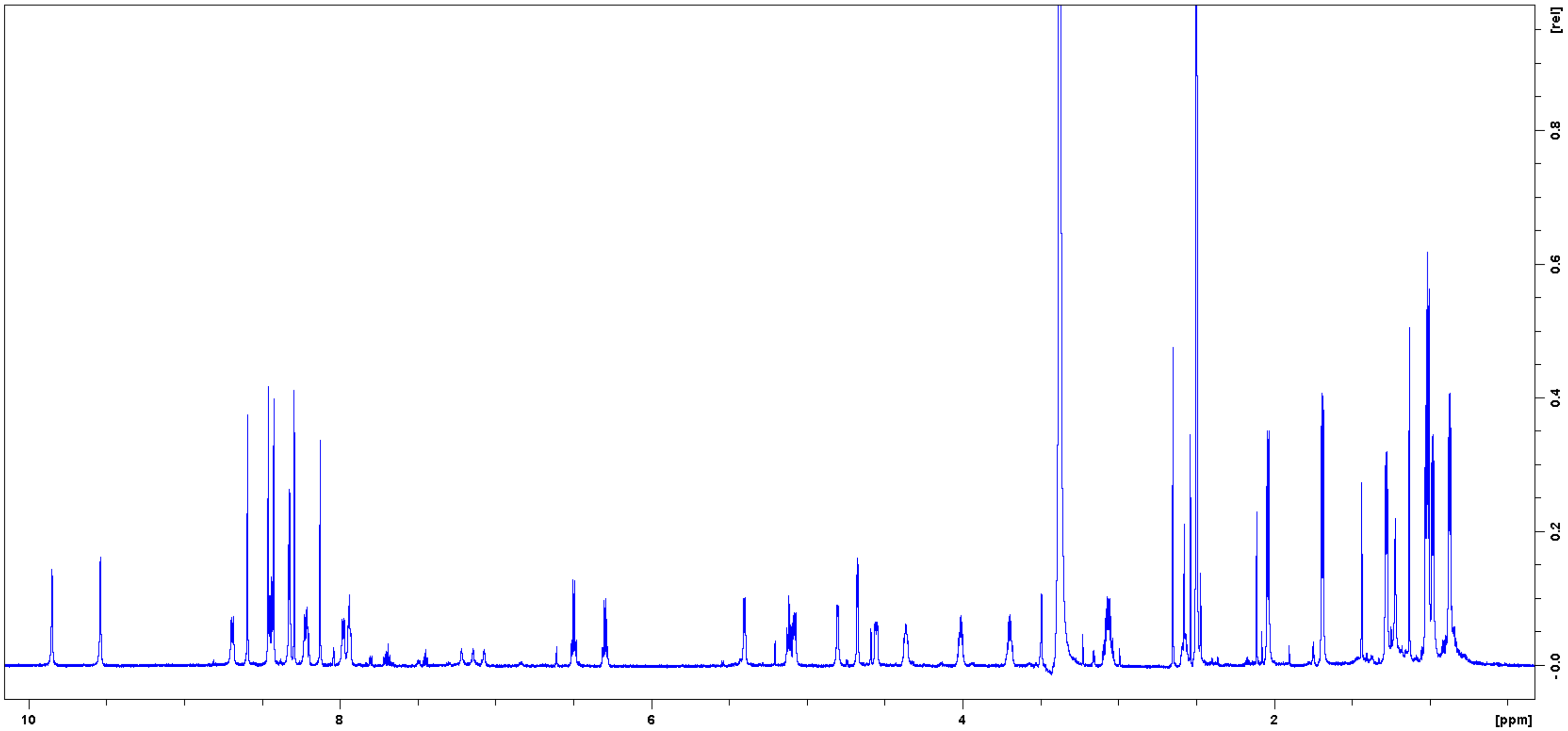

Figure S5. ^{13}C NMR spectrum of micrococcin P3 (**1**)

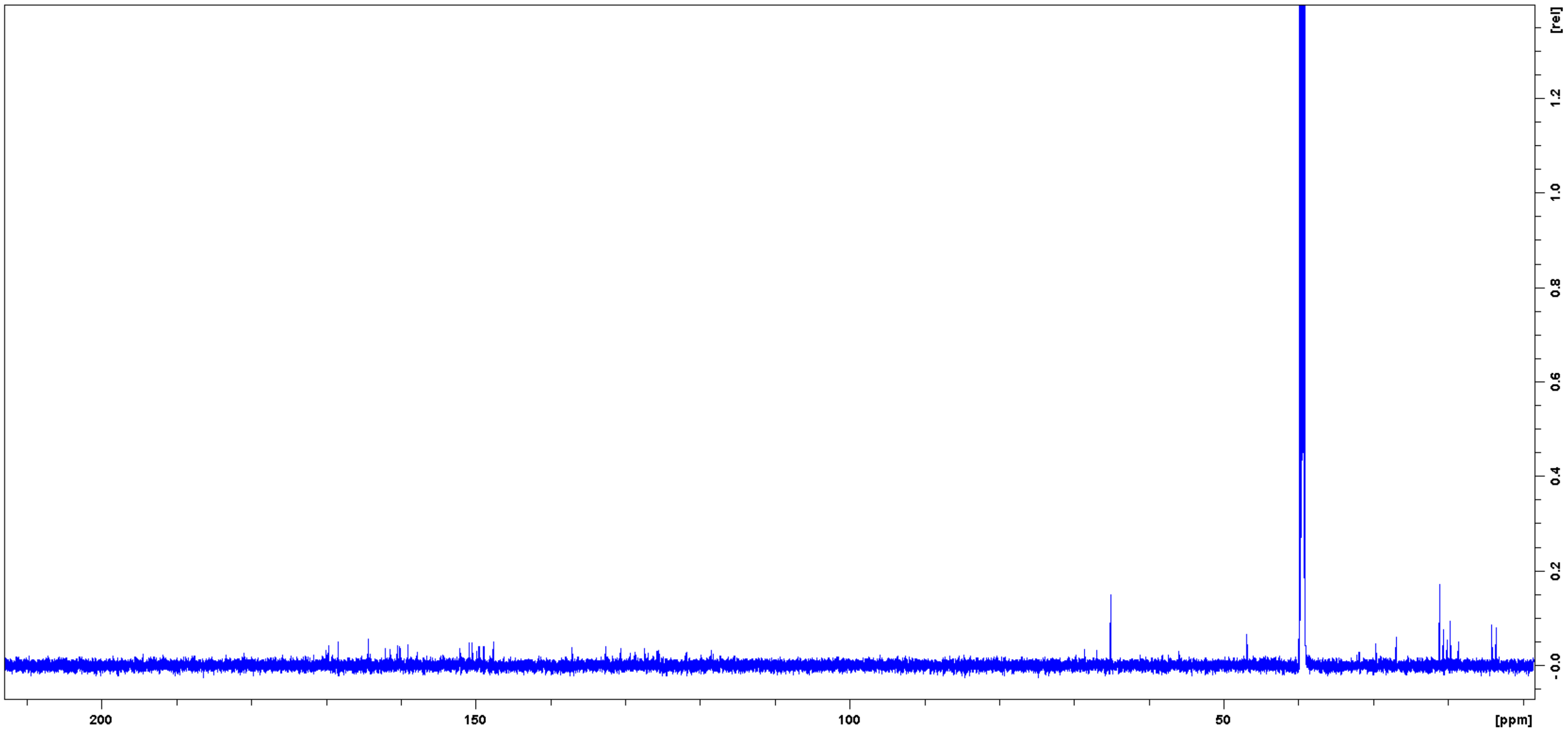

Figure S6. COSY spectrum of micrococcin P3 (**1**)

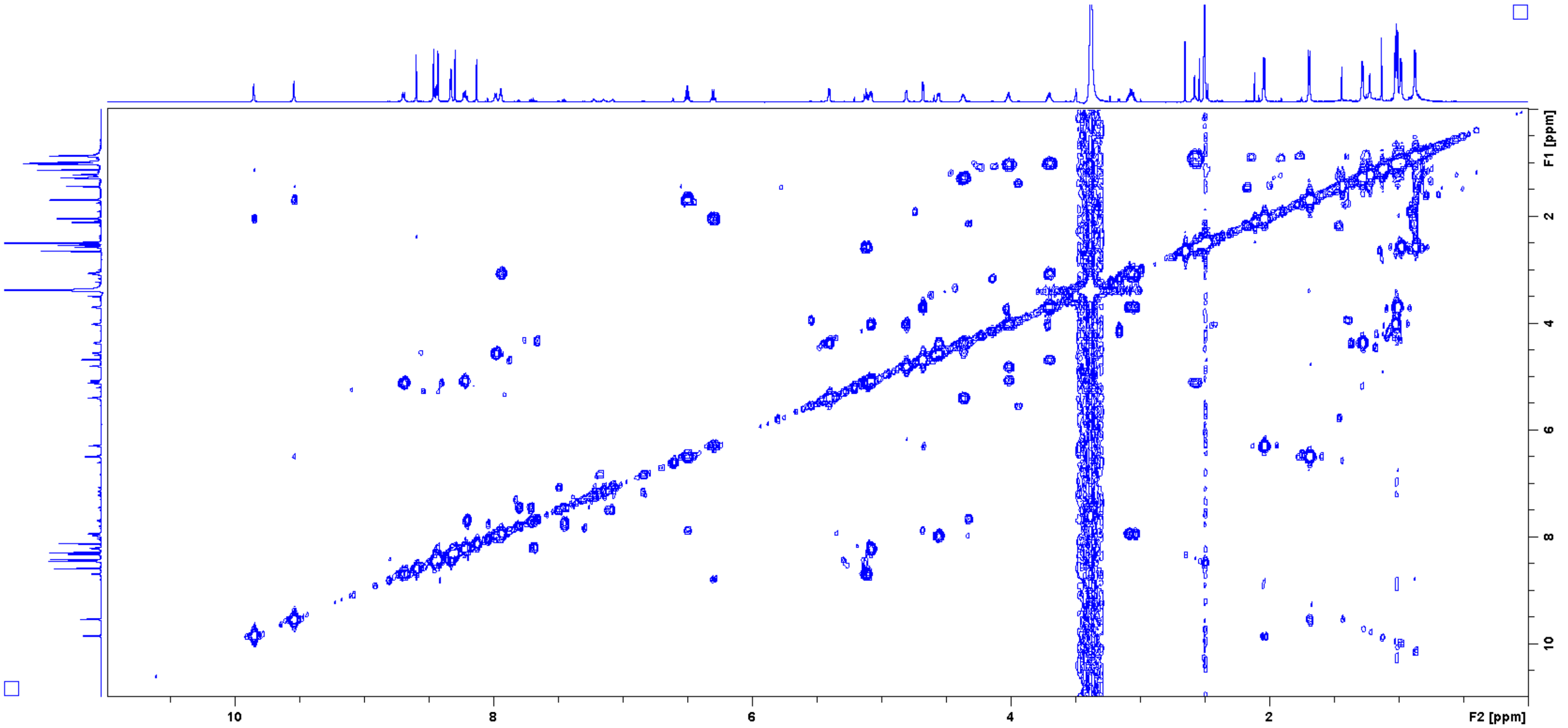

Figure S7. HSQC spectrum of micrococcin P3 (**1**)

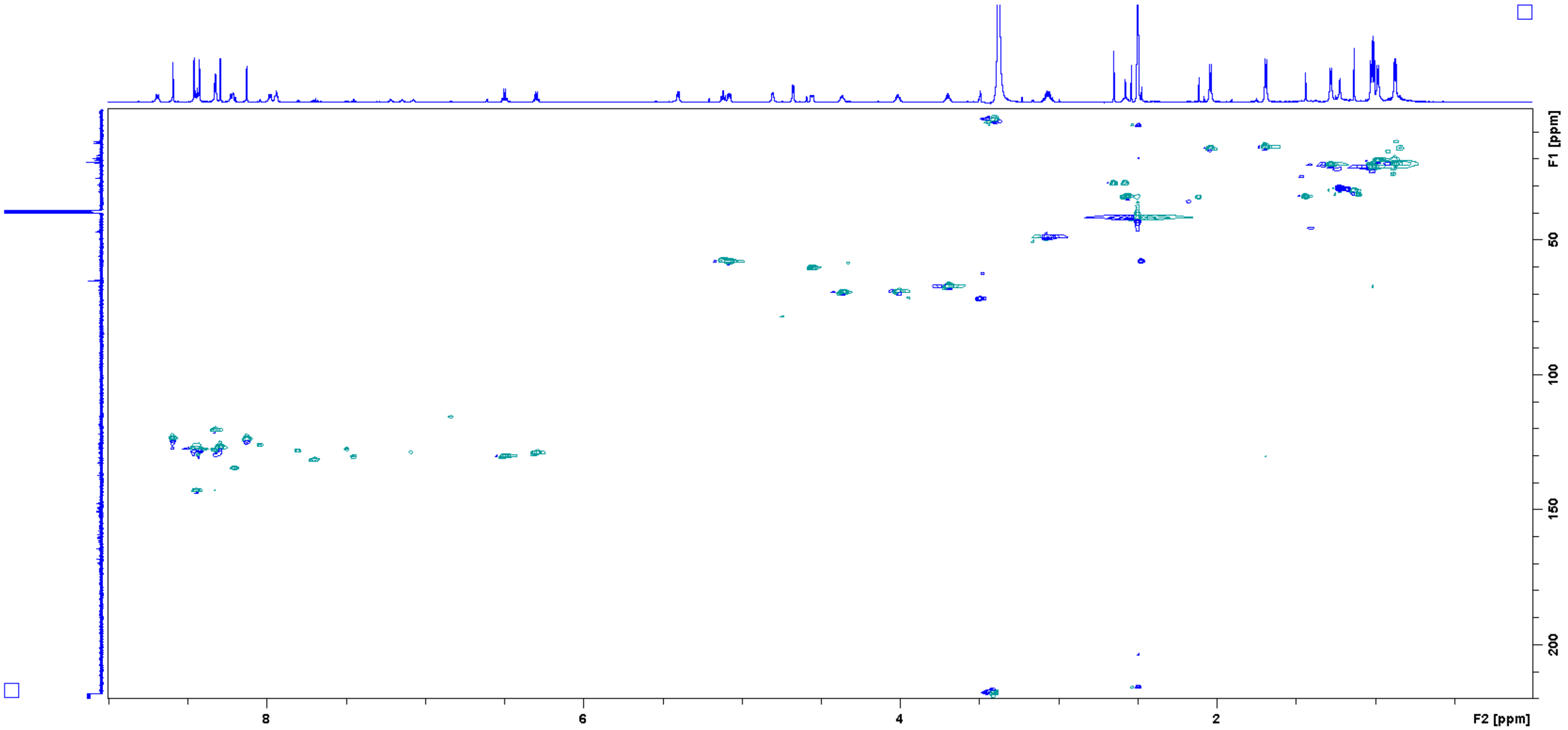

Figure S8. HMBC spectrum of micrococcin P3 (**1**)

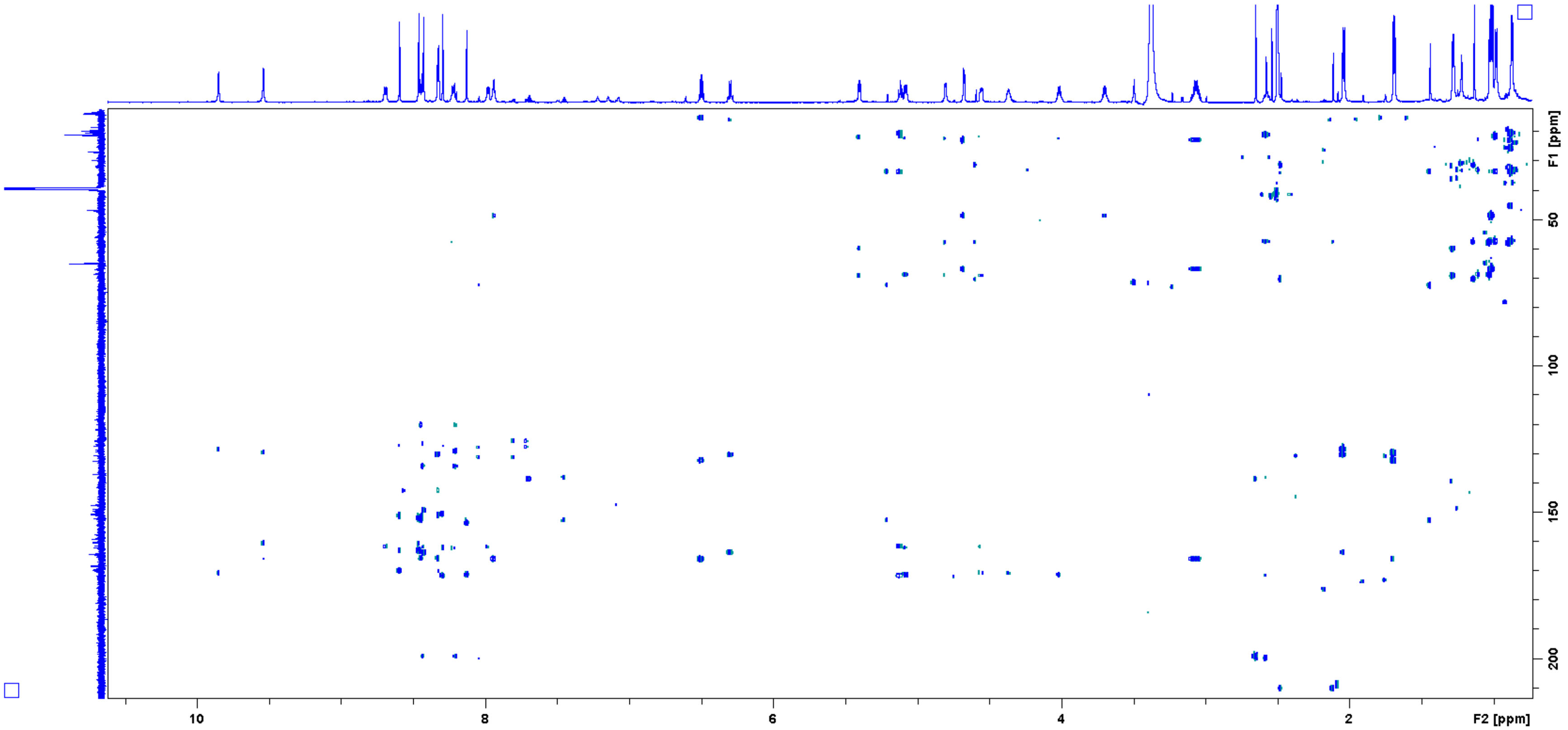

Figure S9. ROESY spectrum of micrococcin P3 (**1**)

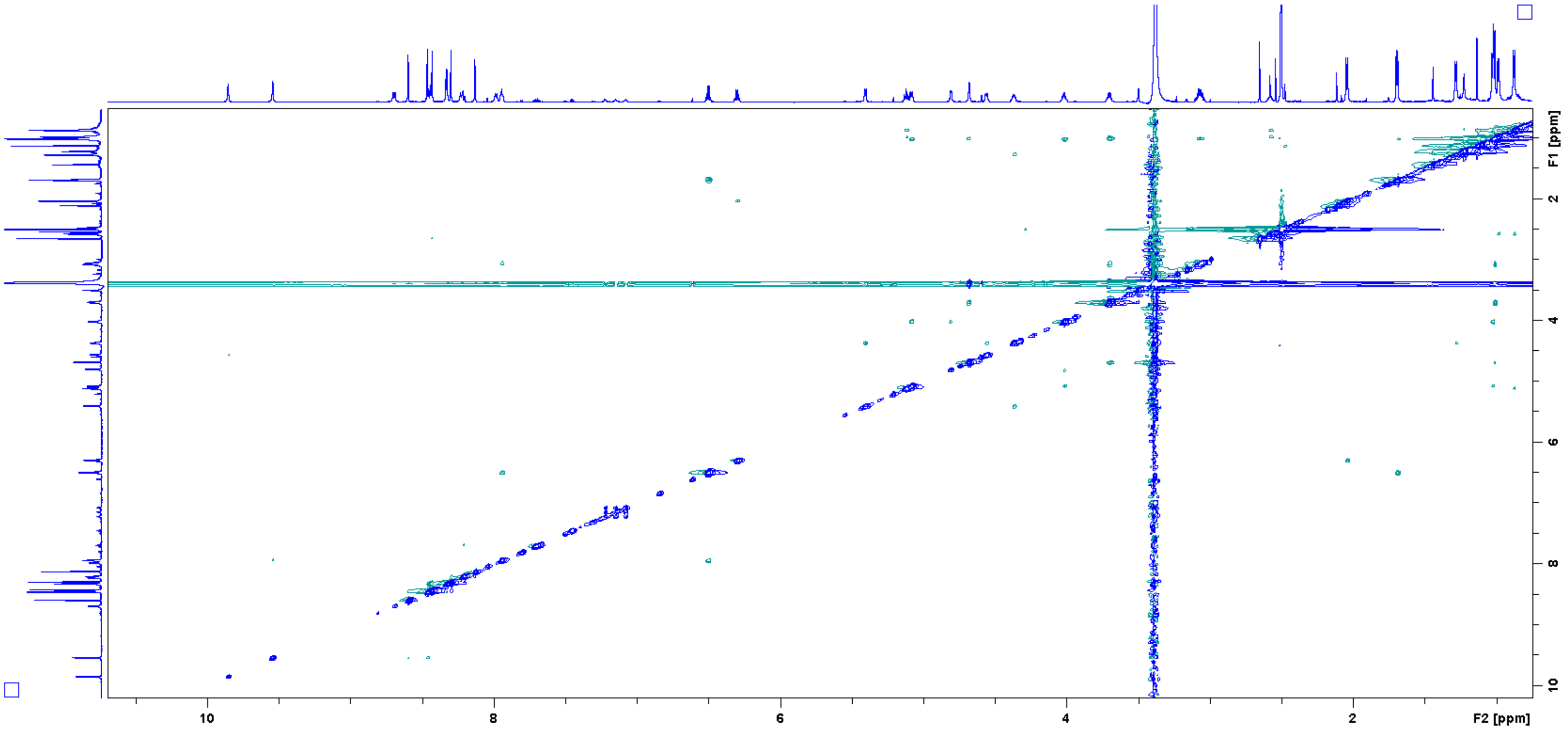

Figure S10. Expended ROESY spectrum of micrococcin P3 (**1**)

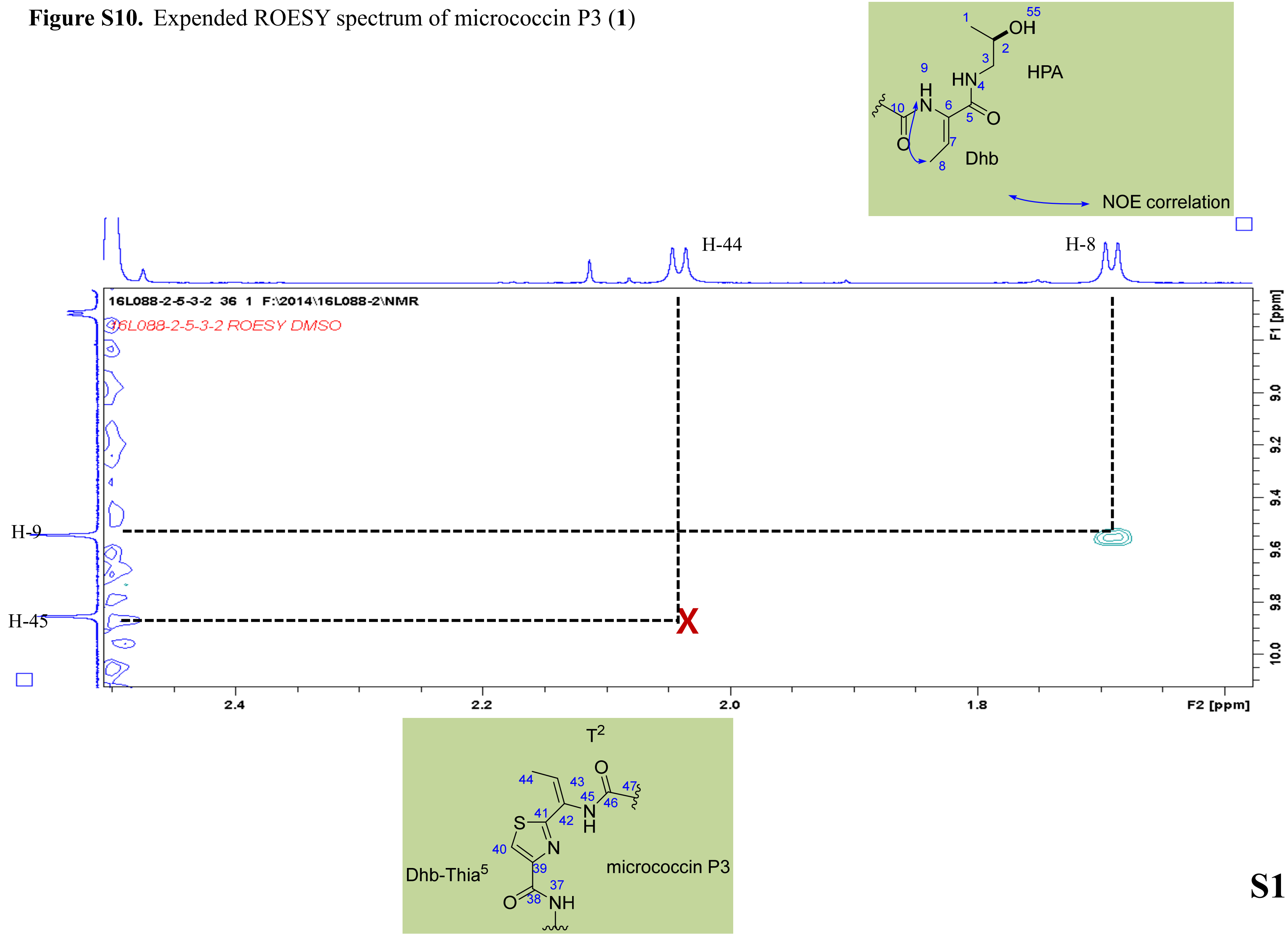

Figure S11. ^{1}H NMR spectrum of micrococcin P1 (**2**)

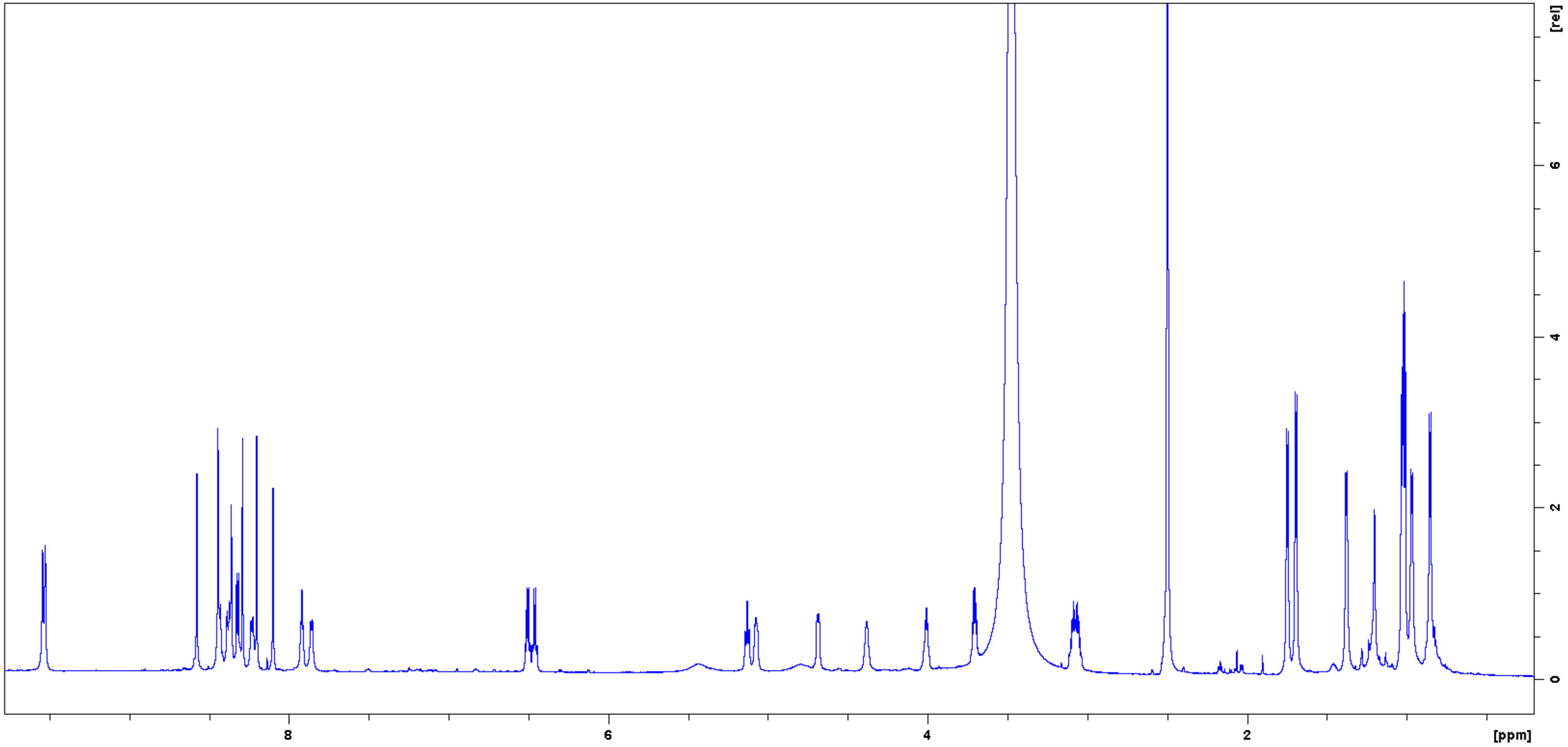

Figure S12. ^{13}C NMR spectrum of micrococcin P3 (**1**)

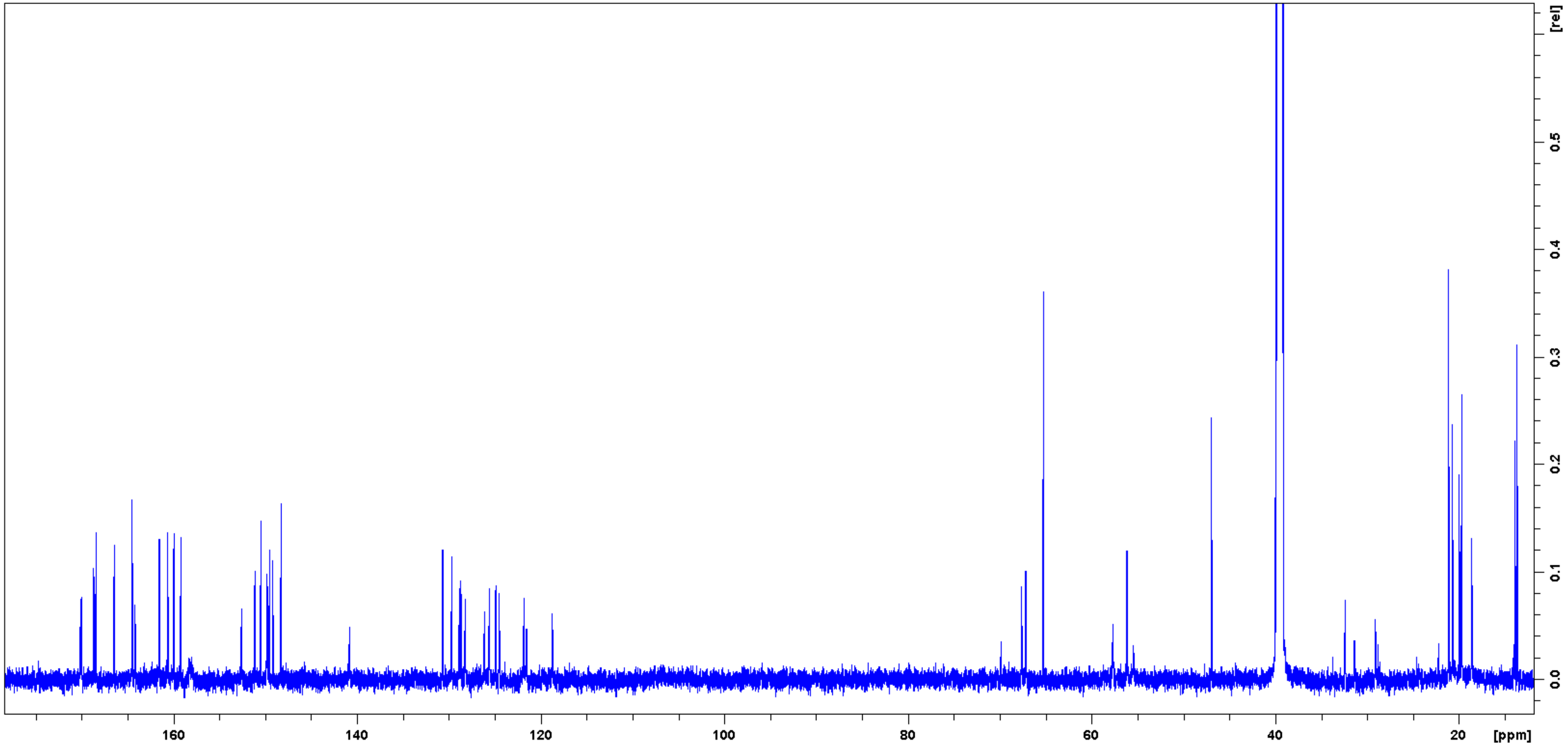

Figure S13. COSY spectrum of micrococcin P3 (**1**)

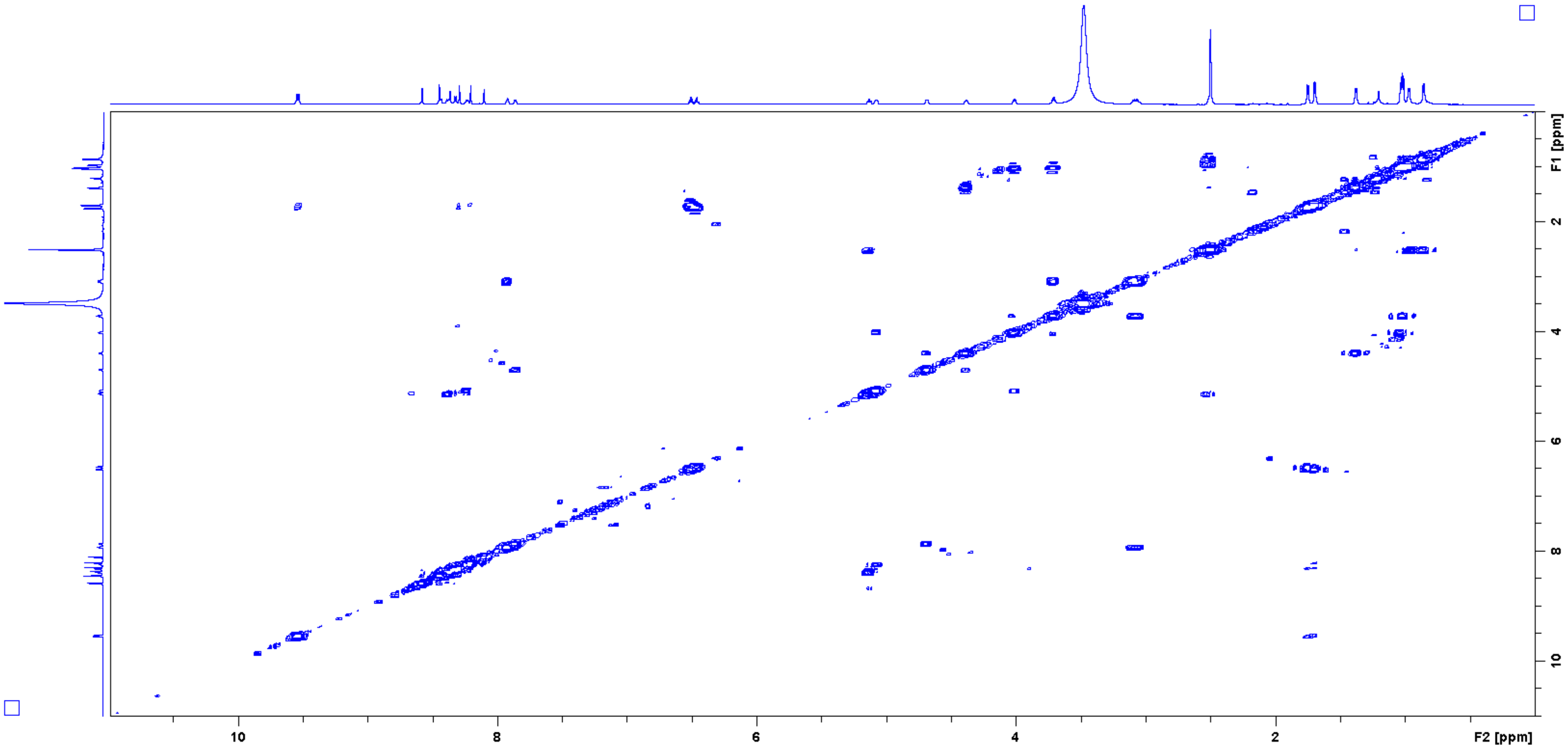

Figure S14. HSQC spectrum of micrococcin P3 (**1**)

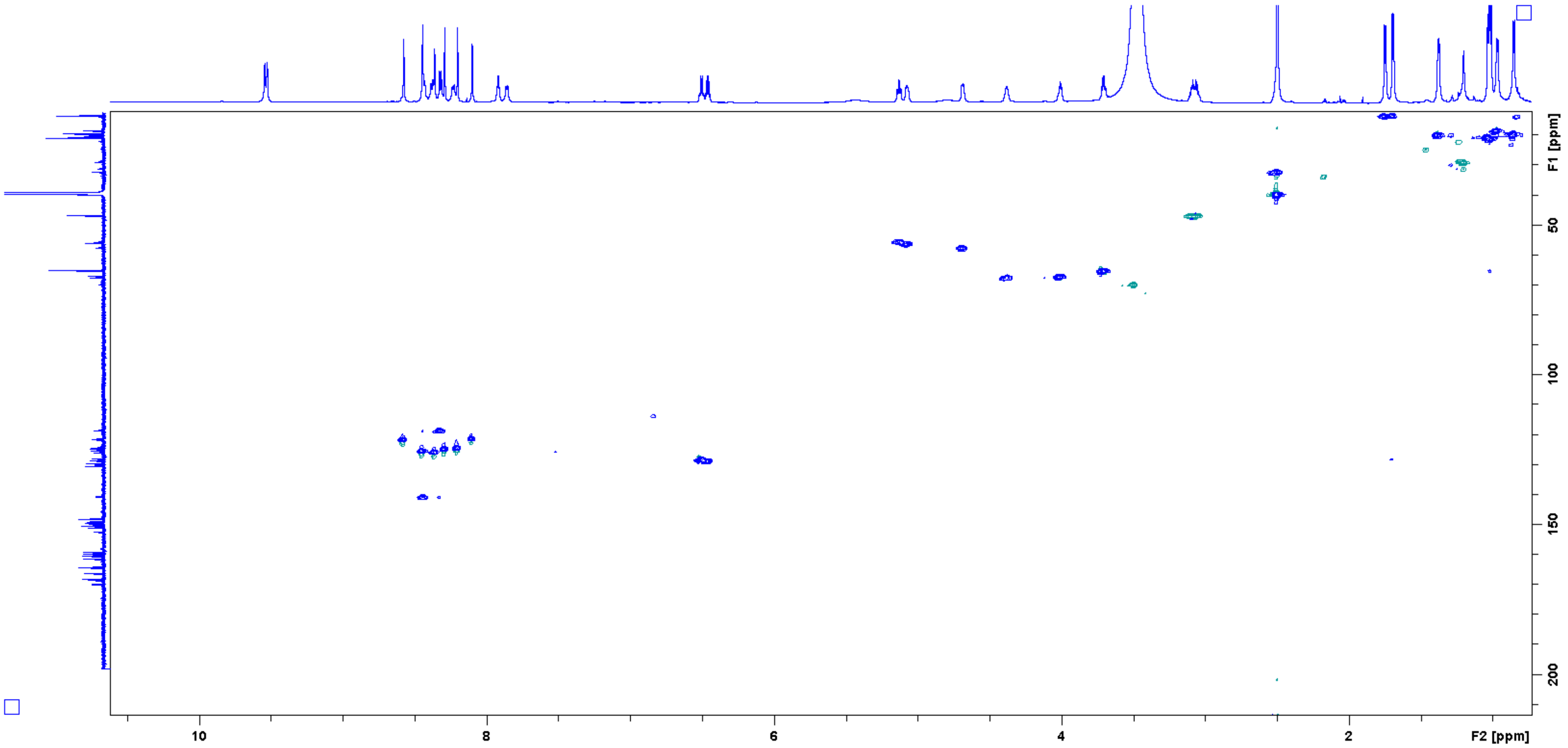

Figure S15. HMBC spectrum of micrococcin P3 (**1**)

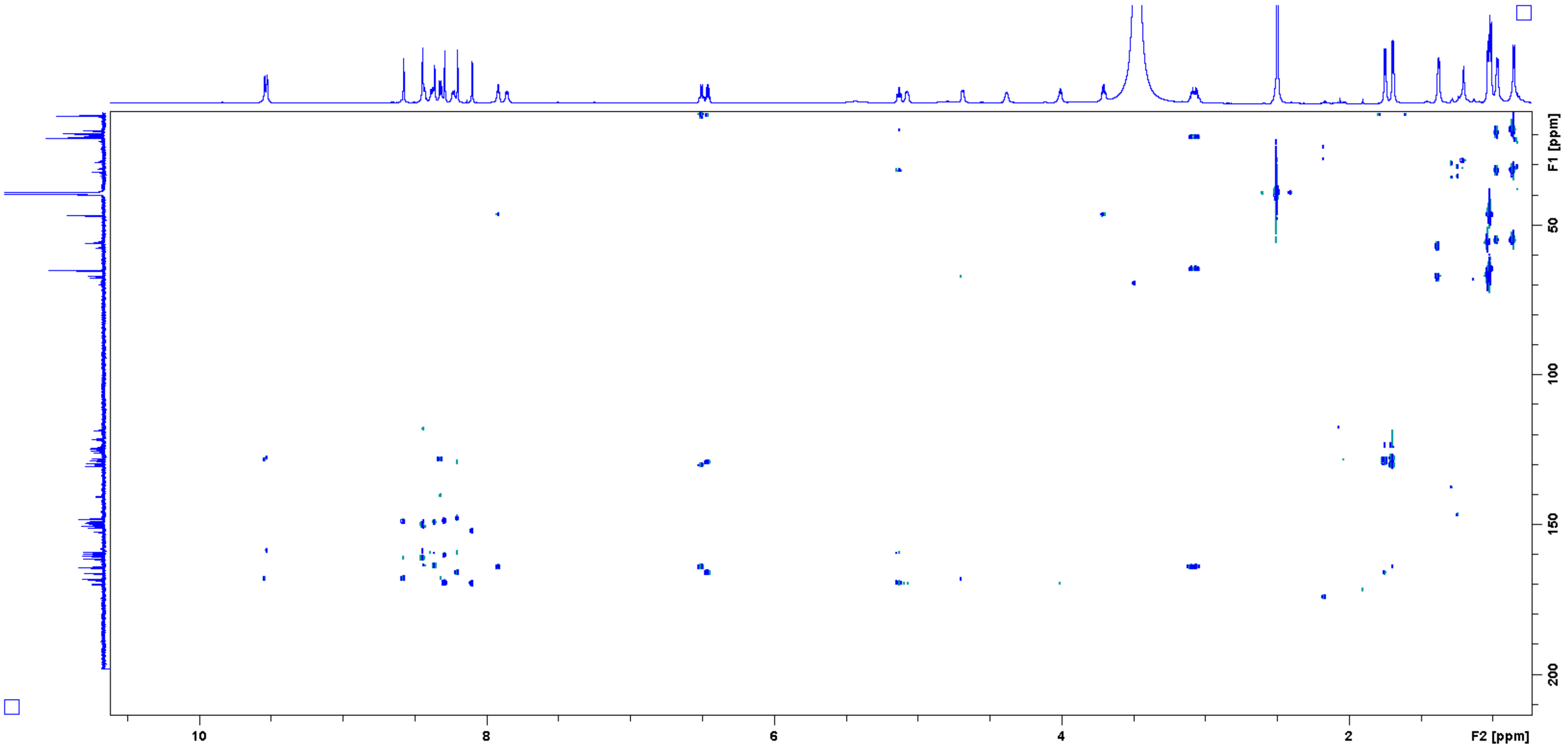

Figure S16. NOESY spectrum of micrococcin P3 (**1**)

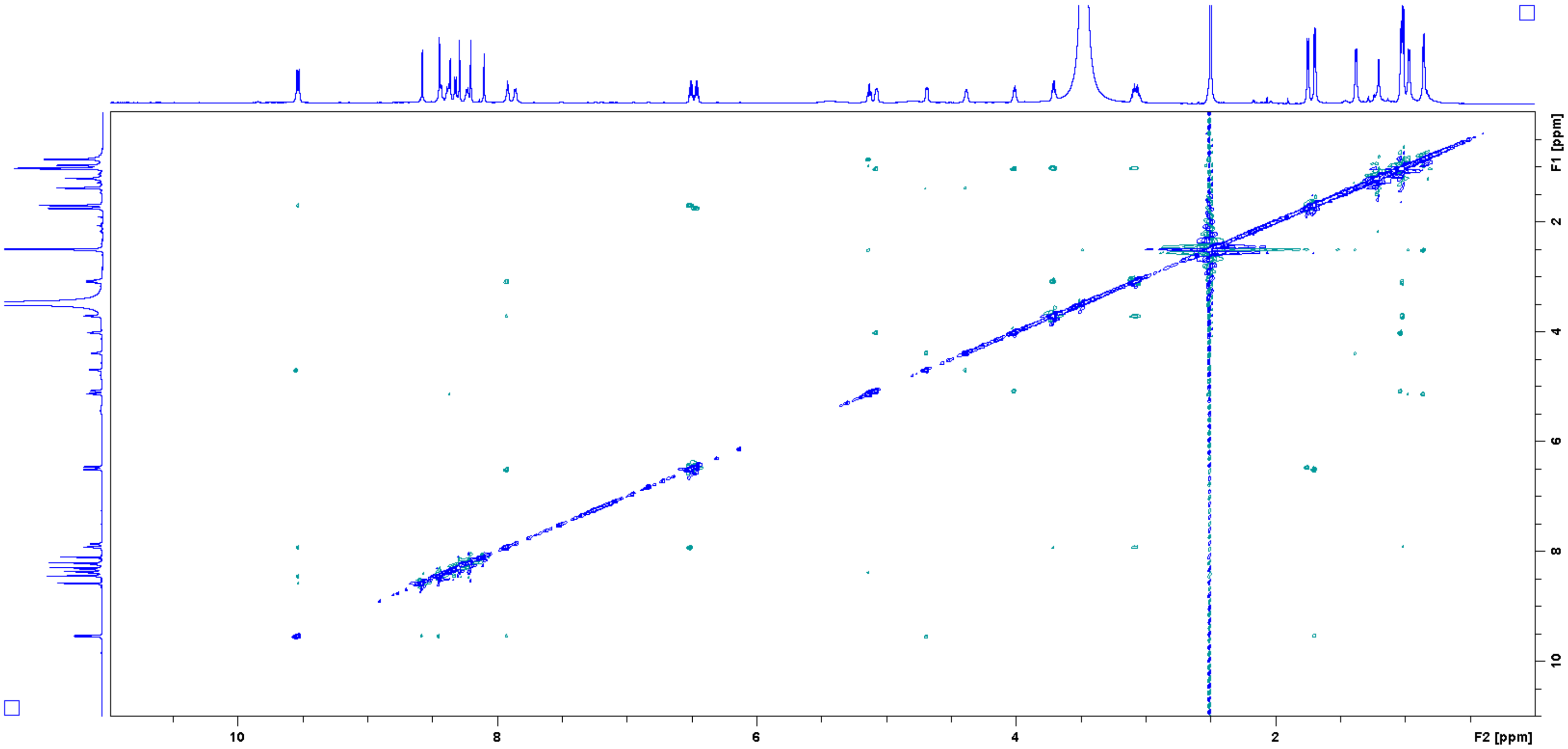

Figure S17. Expended ROESY spectrum of micrococcin P1 (**2**)

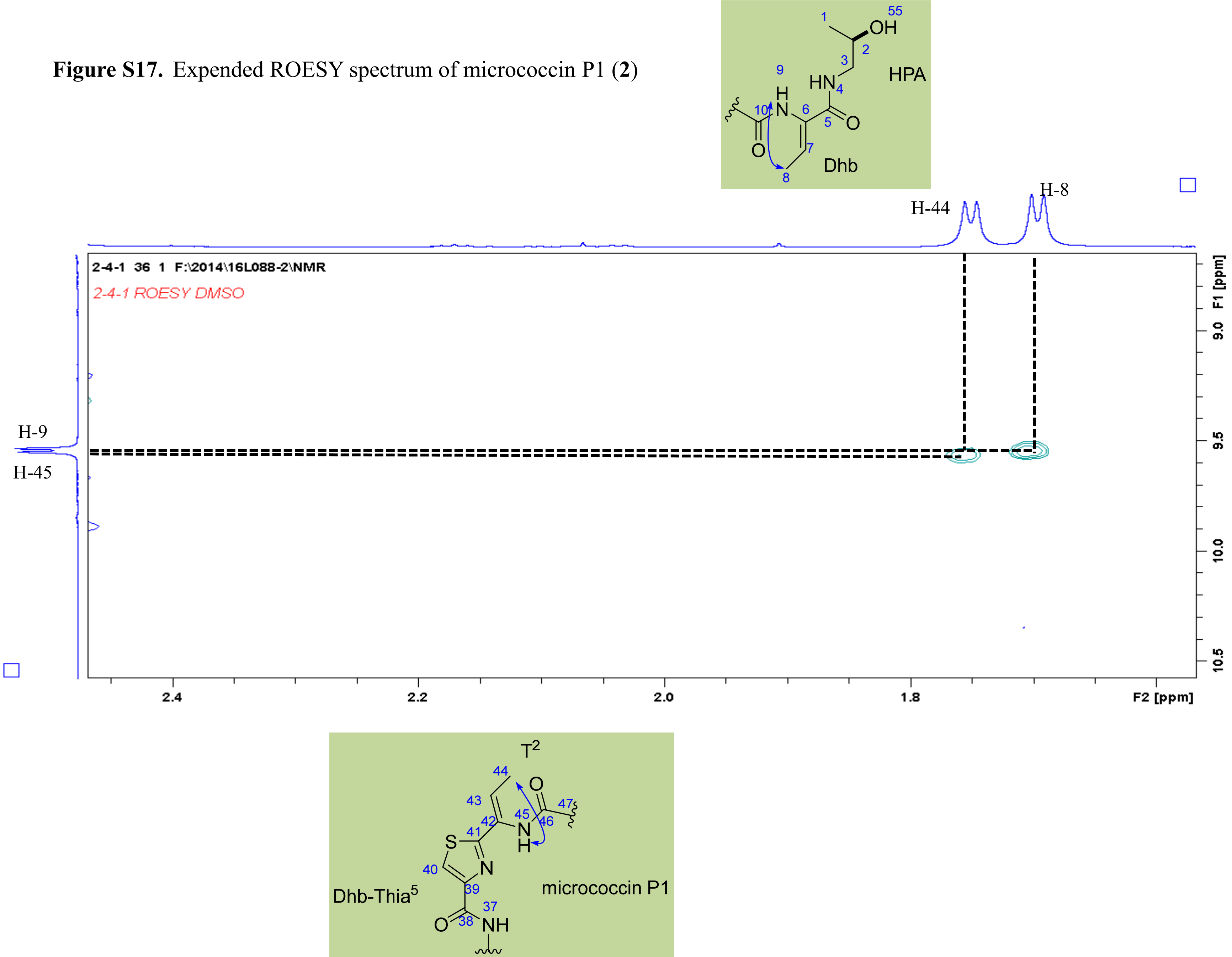

Figure S18. The effects of micrococcins P3 (**1**) and P1 (**2**) on the viability of CV-1 cells

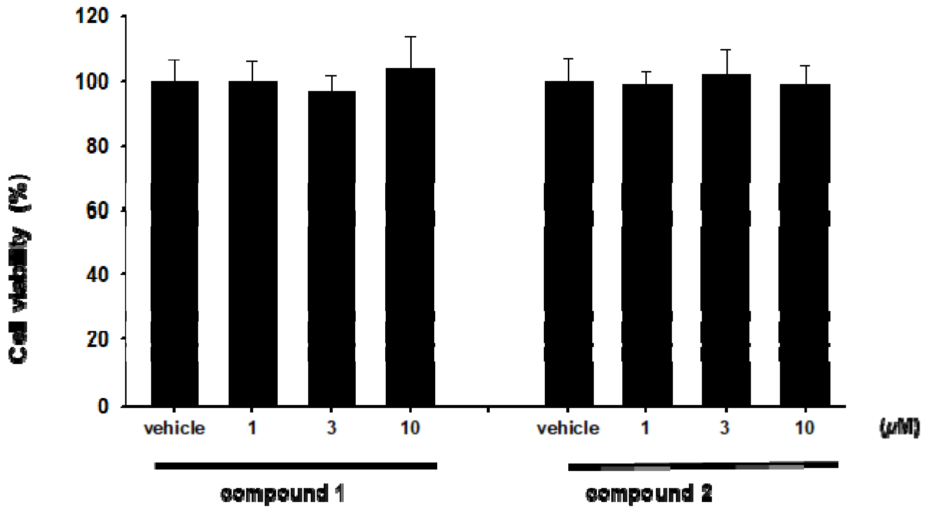

Figure S19. Key MS/MS fragments observed in the spectra of micrococcins P3 (**1**)

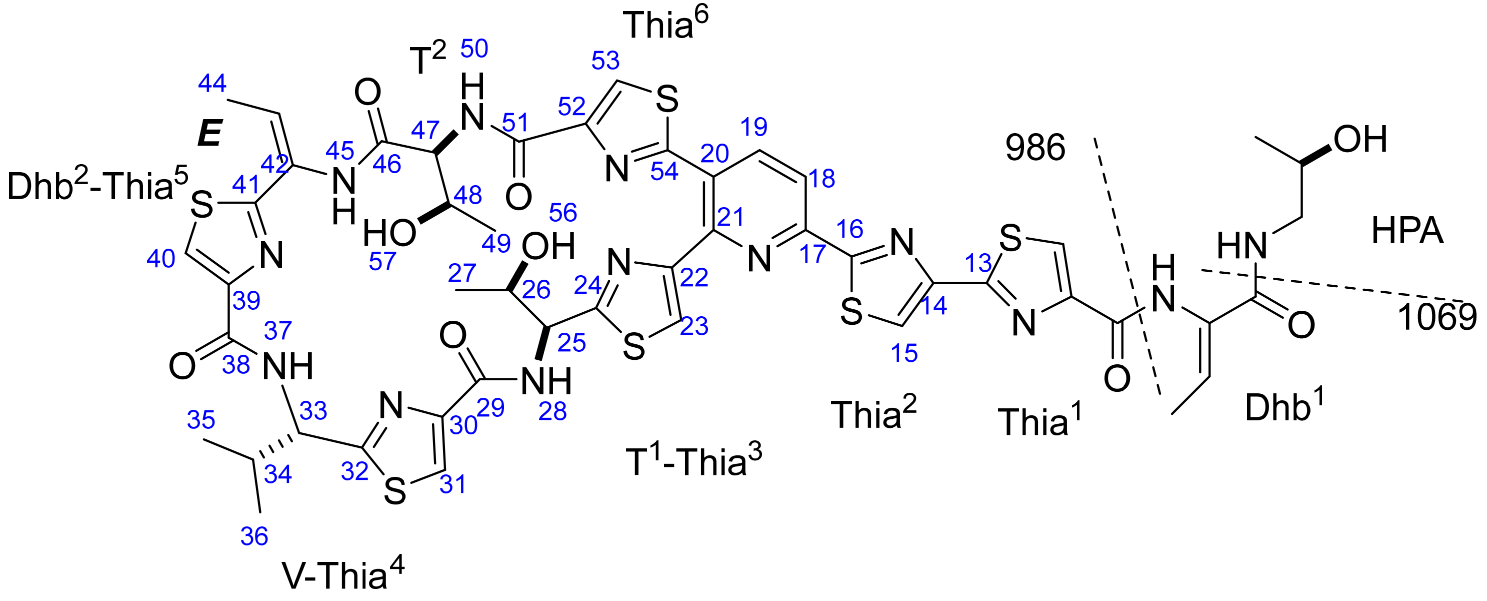

Table S1. Antibacterial activities (MIC μg/mL) of micrococcins P3 (**1**) and P1 (**2**) against marine-derived bacterial strains

marine-derived bacterial strains	Code number of strains	**1**	**2**	Vancomycin	Linezolid	DMSO (v/v)
Shewanella algae	19J07-TSA-3-1	8	8	3.2	1.6	6.3%
Photobacterium damselae	19H09-Blood-2	8	8	3.2	0.8	6.3%
Vibrio parahaemolyticus	19H07-MHAB-S-4	8	8	3.2	0.4	6.3%
Enterococcus faecalis	19J04-blood-1-2	0.5	0.05	1.6	0.1	6.3%
Bacillus amyloliquefaciens_ssp_p lantarum	19H07-D-BHIB-1-2	4	0.5	1.6	1.6	6.3%
Pseudomonas stutzeri	19H07-MHAB-1-2	4	4	0.8	0.8	6.3%

Wells with 8 μg/mL of test compound contained 1.5% DMSO.

Table S2. ^{1}H NMR (700 MHz) data of micrococcin P1 (**2**) in DMSO-d_6 (J in Hz)

No.	**2**	No.	**2**
1	1.02, d (6.2)	31	8.29, s
2	3.71, sext (6.2)	33	5.13, t (9.0)
3	3.08, m	34	2.52, m
4	7.92, t (5.5)	35	0.97, d (6.3)
7	6.51, q (7.0)	36	0.86, d (6.3)
8	1.70, d (7.0)	37	8.38, d (9.0)
9	9.53, s	40	8.20, s
12	8.45, s	43	6.46, q (6.9)
15	8.58, s	44	1.75, d (6.9)
18	8.32, d (8.1)	45	9.54, s
19	8.44, d (8.1)	47	4.69, dd (7.6, 2.9)
23	8.10, s	48	4.38, br s
25	5.08, dd (8.4, 6.2)	49	1.38, d (6.0)
26	4.01, t (6.2)	50	7.86, d (7.6)
27	1.03, d (6.2)	53	8.36, s
28	8.23, d (8.4)		

Table S3. ^{13}C NMR (175 MHz) data of micrococcin P1 (**2**) in DMSO-d_6 (J in Hz)

No.	**2**	No.	**2**
1	21.1	27	20.7
2	65.2	29	160.6
3	46.9	30	149.2
5	164.5	31	124.9
6	130.7	32	170.0
7	128.2	33	55.6
8	13.7	34	32.4
10	159.2	35	18.6
11	150.5	36	19.7
12	125.6	38	159.9
13	161.5	39	148.3
14	149.5	40	124.5
15	121.8	41	166.4
16	168.4	42	130.0
17	149.8	43	128.8
18	118.7	44	13.9
19	140.8	46	168.7
20	128.7	47	57.7
21	151.1	48	67.6
22	152.6	49	20.0
23	121.5	51	159.9
24	170.1	52	149.7
25	56.1	53	126.1
26	67.2	54	164.2
